# Genital tuberculosis in a tamoxifen-treated postmenopausal woman with breast cancer and bloody vaginal discharge

**DOI:** 10.1186/1476-0711-5-20

**Published:** 2006-09-01

**Authors:** Ioannis Neonakis, Elpis Mantadakis, Zoe Gitti, Ioanna Mitrouska, Louis George Manidakis, Sofia Maraki, George Samonis

**Affiliations:** 1 Laboratory of Mycobacteriology, Department of Clinical Bacteriology, Parasitology, Zoonoses, and Geographical Medicine, University Hospital of Heraklion, 712 01 Heraklion, Crete, Greece; 2 Department of Internal Medicine, University Hospital of Heraklion, 712 01 Heraklion, Crete, Greece; 3 Department of Thoracic Medicine, University Hospital of Heraklion, 712 01 Heraklion, Crete, Greece; 4 Department of Obstetrics and Gynecology, University Hospital of Heraklion, Heraklion, Crete, Greece

## Abstract

**Background:**

Female genital tuberculosis is an uncommon disease that is rarely diagnosed in developed countries.

**Case presentation:**

A 61-year-old postmenopausal woman who had undergone surgery and treated with adjuvant chemotherapy for infiltrating ductal carcinoma of the breast five years ago, presented with bloody vaginal discharge, fatigue, weight loss, and low grade fevers at night for two months. Histological examination of the endometrium, done based on the suspicion of a second primary cancer due to the tamoxifen therapy, revealed a granulomatous reaction. Liquid and solid mycobacterial cultures of the tissues were performed. Although the acid fast staining was negative, the liquid culture was positive for *Mycobacterium tuberculosis*. Involvement of other systems was not detected. The patient was treated with a three-drug antituberculosis regimen for 9 months and recovered fully.

**Conclusion:**

Female genital tuberculosis is a rare but curable disease that should be included in the differential diagnosis of women with menstrual problems. Early diagnosis is important and may prevent unnecessary invasive procedures for the patient.

## Background

Tuberculosis (TB) predominately presents with pulmonary disease, although extra-pulmonary TB is not uncommon [1-4]. Diagnosis of extra-pulmonary TB is often difficult, because of its non-specific clinical and laboratory findings [2]. Female genital TB (FGTB) is usually a symptom-less disease diagnosed during investigations for infertility [5-7]. Symptomatic disease usually presents with pelvic pain or menstrual irregularities [8,9]. FGTB is extremely rare in postmenopausal women [10]. We present a case of FGTB in a tamoxifen-treated postmenopausal woman with breast cancer and bloody vaginal discharge.

## Case presentation

A 61-year-old postmenopausal woman diagnosed in the past with breast cancer presented to her Oncologist for regular follow up. The patient complained of fatigue, weight loss, persistent slight evening pyrexia, a mild lower abdominal pain for the last two months, along with leukorrhea and bloody vaginal discharge for the last three weeks. Five years ago, during the perimenopausal period, she was diagnosed with an infiltrating ductal carcinoma of the breast. She underwent lumpectomy followed by adjuvant chemotherapy and radiotherapy. Since then she was receiving oral tamoxifen that she was due to stop in one month and had no clinical, laboratory or radiological evidence of recurrent or metastatic disease.

Physical examination revealed mild abdominal distension and an enlarged, tense, and tender uterus. A chest radiograph was negative, while a full blood count, serum electrolytes, and liver function tests were normal. A transvaginal ultrasonogram showed an enlarged uterus with a 7 mm endometrial thickness (normal for age up to 4 mm). Hysteroscopy and endometrial aspiration biopsy performed because of the suspicion of a second primary cancer of the endometrium from the tamoxifen therapy disclosed necrotic debris with an extensive granulomatous reaction, numerous tubercles composed of epithelioid cells, and multinucleated giant cells with no central necrosis. These findings raised the suspicion of FGTB. Hence, samples from the endocervix and the endometrium were obtained with the use of a sterile Cyto Brush for PAP smear, suspended in sterile water, and processed according to standard guidelines [11] followed by cultures in solid (Lowenstein-Jensen, BioMerieux, France) and liquid media (BacT/Alert 3D, Organon Teknika, NC). Although acid-fast staining was negative, the liquid culture gave a positive signal after 7 days of incubation. The presence of *Mycobacterium tuberculosis *was verified by AccuProbe assay (Gen-Probe, CA) and phenotypic-biochemical analysis. Since the samples submitted for culture from the endocervix and the endometrium were not separated, it is unclear if TB was limited to the endocervix, the endometrium or both.

Tuberculin skin testing was positive (>15 mm), while detailed family history revealed that the patient's mother and sister had died 30 and 10 years ago from TB. The patient had taken close care of both. No other family members had symptoms suspicious of TB. Interestingly, the patient had refused this family history at presentation to her Oncologist. Finally, she admitted the contact history to patients infected with TB only after the results of the cultures were available. In order to rule out pulmonary and renal spread of the disease, imaging and laboratory evaluation with chest radiograph and examination of multiple early morning sputum and urine samples with acid-fast staining and cultures was performed and showed no lung or renal involvement.

Drug susceptibility testing was performed by the proportional methods on Lowenstein-Jensen medium. The isolated strain was resistant to pyrazinamide (200 μg/ml), streptomycin (4 μg/ml), ethionamide (30 μg/ml), rifampicin (20 μg/ml) and pefloxacine (25 μg/ml) and susceptible to isoniazid (0.2 μg/ml), rifampicin (40 μg/ml), ethambutol (2 μg/ml), streptomycin (10 μg/ml) and para-4-aminosalicylic acid (0.5 μg/ml). The patient was treated with a three-drug regimen of isoniazid (300 mg/day), rifampicin (600 mg/day) and ethambutol (1500 mg/day) for nine months. Within a month after the beginning of antituberculosis therapy, the vaginal discharge disappeared along with resolution of the fatigue, a two kilogram weight gain, and complete resolution of the persistent evening pyrexia and the lower abdominal pain.

## Discussion

Despite the remarkable efforts to control TB worldwide over the past decades, almost two million people still die of this disease every year, justifying the declaration of TB as a public health emergency by the 44^th ^World Health Assembly. FGTB is an uncommon presentation. Its exact incidence remains unknown, as the majority of the cases remain undiagnosed. In Western countries the incidence of FGTB is estimated to be <1%, whereas in some African and Asian countries it reaches 15–19% [12,13].

Although in some cases cervical TB is thought to be the primary infection [14,15], FGTB is usually a secondary complication of pulmonary TB. In most cases it begins with a focus in the endosalpinx and spreads to the endometrium, the ovaries, occasionally to the cervix and rarely the vagina [8]. In our case it is unclear whether the disease was limited to the endocervix and/or the endometrium since the samples provided for culture were not separated.

In the developing world, almost nine out of ten cases are young women between 20 and 40 years of age [16], whereas, in Western countries it is usually diagnosed in women over the age of 40, usually pre-menopausal ones [17-19]. The atrophic endometrium of postmenopausal women is thought to offer a poor milieu for the growth of *M. tuberculosis *[10]. Hence, in contrast to premenopausal women where infertility is the main symptom leading the suspected gynecologist to seek for FGTB, in postmenopausal women vaginal bleeding is the usual presenting symptom of the disease.

FGTB is rarely diagnosed in developed countries. Symptoms are usually absent and there is often no evidence of TB in other organs. If present, the symptoms are mild and local, such as abdominal pain or menstrual irregularities. Infertility is the most common consequence [7]. Due to its rarity and its mild clinical picture, the index of suspicion for the diagnosis of FGTB among gynecologists is usually low. This is unfortunate since early detection and proper treatment can, in some cases, prevent irreversible damage to the fallopian tubes and the endometrium. Once fibrosis is established, fertility is generally difficult to restore even with appropriate treatment [9]. Nevertheless, early diagnosis can save the patient from a series of invasive diagnostic or therapeutic procedures. To this end, identification of high risk patients is helpful. Suspicions should be raised if a woman has a personal history of latent tuberculous infection or previous contact with persons suffering from TB. In the present case, persistent questioning revealed the patient's prior close contact with infected persons. Furthermore, the clinician should be suspicious if the patient originates from areas where TB or AIDS are endemic, since more than 3% of new cases of TB are related to HIV infection [13]. Additionally, in some developing countries, as much as 60–90% of extrapulmonary TB occurs in HIV positive patients [13,20]. Since genital disease often causes no physical symptoms, infertile women should be examined for TB, especially in case of predisposing factors. This is also true for post-menopausal women with vaginal bleeding. Samples from the endometrium and the endocervix should be submitted for cytological examination, acid fast stains, and cultures, since combination of different methods is likely to have a higher diagnostic yield for TB.

The diagnostic role of a positive Mantoux (purified protein derivative-PPD) skin test is controversial. In a recent study, almost 45% of infertile women with strong indirect evidence of pelvic TB, such as laparoscopic findings (thickened tubes, areas of caseation, etc.), had a negative PPD skin test [21]. On the other hand, in a group of 27 infertile women with a positive PPD skin test, only 11 had clear laparoscopic findings suggestive of FGTB. The other conventional diagnostic methods of TB, such as chest radiographs, urine, and sputum cultures are not specific for FGTB, but are helpful to rule out dissemination to other organs. Infertile women with a positive PPD skin test should undergo early laparoscopy for direct visualization of the fallopian tubes and for collection of material from the pelvis for pathological examination and cultures [21]. Laparoscopic findings, such as the presence of excessive peritoneal fluid, rigid and almost immobile tubes, presence of tubercles or caseous material are indicative but not pathognomonic of FGTB. The value of other diagnostic means, such as sonography or hysterosalpingography remains controversial [19].

Histological detection of typical granulomata is sufficient for diagnosis of pelvic TB, providing that all other causes of granulomatous reactions have been ruled out. Differential diagnosis includes tularemia, sarcoidosis, brucellosis, amoebiasis, schistosomiasis and foreign body reactions [9,22]. Identification of extragenital TB strongly supports the diagnosis. Nevertheless, the gold standard for diagnosis of FGTB is the isolation of *M. tuberculosis *from appropriate specimens, usually biopsy materials from the endometrium. Samples can be used for the detection of *M. tuberculosis *DNA by PCR or can be cultured [16]. Efforts are currently made to use menstrual blood, when available, for molecular or serological diagnosis [16].

In the present case, the differential diagnosis included a second primary cancer of the endometrium due to the prolonged use of tamoxifen and possible genital TB or other granulomatous diseases. The use of a Cyto Brush was effective for proper sample taking, while isolation of *M. tuberculosis *was achieved by conventional methods. The use of Cyto Brush has some advantages compared with biopsy, such as simplicity, ease of use, low cost, and minimal patient discomfort. To our knowledge, no studies have directly compared biopsy versus Cyto Brush or Cyto Brush versus other conventional sampling methods, e.g., wooden spatulas or cotton swabs for isolation of mycobacteria, although the hydrophobic nature of the lipid-containing cell wall of mycobacteria inhibits their transfer from the cotton fibers to the aqueous culture medium [23].

Acid-fast staining was negative in our patient. This test is infrequently positive, since approximately 10^5 ^mycobacteria per milliliter are required in order to have a positive result [19]. On the other hand, liquid mycobacterial cultures are useful, usually leading to isolation within days. Nowadays, a series of automatic liquid culture systems are available contributing to faster diagnosis.

In conclusion, FGTB is a rare but curable disease that should be included in the differential diagnosis of postmenopausal vaginal bleeding. Early diagnosis is important and it can save the patient from unnecessary invasive procedures.

## Abbreviations

TB: tuberculosis, FGTB: Female genital tuberculosis, PPD: purified protein derivative

## Competing interests

The author(s) declare that they have no competing interests.

## Authors' contributions

IN carried out the laboratory procedures and drafted the paper. EM helped to draft the manuscript and revised it critically. ZG participated in carrying out the laboratory procedures. IM handled the patient's antituberculosis treatment. LGM performed the hysteroscopy and endometrial aspiration biopsy of the patient. SM participated in the coordination of the clinical and laboratory work-up. GS conceived the report, treated the patient for the breast cancer, revised the manuscript and gave final approval of the submitted version. All authors have read and approved the final manuscript.
